# A Novel Passive Tracking Scheme Exploiting Geometric and Intercept Theorems

**DOI:** 10.3390/s18030895

**Published:** 2018-03-17

**Authors:** Biao Zhou, Chao Sun, Deockhyeon Ahn, Youngok Kim

**Affiliations:** 1 The School of Internet of Things (IoT) Engineering, Jiangnan University, Wuxi 214122, China; zhoubiao@jiangnan.edu.cn; 2 Electronic Engineering Department, Kwangwoon University, Seoul 01897, Korea; sunchao2601@gmail.com (C.S.); ejrgus456@gmail.com (D.A.)

**Keywords:** radio frequency tomography, passive tracking, geometric theorems, intercept theorem

## Abstract

Passive tracking aims to track targets without assistant devices, that is, device-free targets. Passive tracking based on Radio Frequency (RF) Tomography in wireless sensor networks has recently been addressed as an emerging field. The passive tracking scheme using geometric theorems (GTs) is one of the most popular RF Tomography schemes, because the GT-based method can effectively mitigate the demand for a high density of wireless nodes. In the GT-based tracking scheme, the tracking scenario is considered as a two-dimensional geometric topology and then geometric theorems are applied to estimate crossing points (CPs) of the device-free target on line-of-sight links (LOSLs), which reveal the target’s trajectory information in a discrete form. In this paper, we review existing GT-based tracking schemes, and then propose a novel passive tracking scheme by exploiting the Intercept Theorem (IT). To create an IT-based CP estimation scheme available in the noisy non-parallel LOSL situation, we develop the equal-ratio traverse (ERT) method. Finally, we analyze properties of three GT-based tracking algorithms and the performance of these schemes is evaluated experimentally under various trajectories, node densities, and noisy topologies. Analysis of experimental results shows that tracking schemes exploiting geometric theorems can achieve remarkable positioning accuracy even under rather a low density of wireless nodes. Moreover, the proposed IT scheme can provide generally finer tracking accuracy under even lower node density and noisier topologies, in comparison to other schemes.

## 1. Introduction

The positioning technique of adopting received signal strength (RSS) plays a vital role in wireless sensor networks (WSNs), because the RSS is ubiquitous in radio frequency (RF) environments and can be easily acquired without extra hardware costs. According to the application scenarios in WSNs, the state-of-the-art of RSS-based positioning systems fall into two categories [1,2]: active positioning schemes and passive tracking schemes. For active positioning schemes, the target is supposed to carry wireless-assisted equipment, such as RF tags, smartphones, wearable devices, or laptops, to interact with the anchor nodes in the WSNs. Then, the target can be located by measuring the RSS information between the assisted wireless equipment and the anchor nodes [3,4,5,6].

In many cases, however, the targets are not expected to carry any effective devices for assisting the positioning work. For instance, intruders always avoid holding devices that can communicate with monitoring systems, and the handset device of an uninvited guest may not be able to provide RSS information estimating its trajectory to the monitoring system. Moreover, a kidnapped person or a child cannot use any wireless devices. The targets under such circumstances are defined as device-free targets, and capturing the position information of device-free targets is termed as a passive tracking problem, also known as device-free localization [1,2,7,8,9,10,11,12,13,14,15,16].

Indoor passive tracking in the WSN environment using RF signal processing was introduced by M. Youssef et al. in Reference [1]; they describe device-free localization systems architecture as well as challenges that need to be addressed for a feasible passive tracking system. Recently, one of the most popular principles for RSS-based passive tracking has been based on RF Tomography technology. In a broad sense, when a target moves into an area monitored by a group of line-of-sight links (LOSLs) between pairs of wireless nodes, there would be an RSS fluctuation between these pairs of nodes, which is termed the phenomenon of RF Tomography [7,8,9]. In References [10,11], J. Wilson et al. carried out RF tomography imaging for device-free tracking for the first time; since then, a great deal of research has focused on applying RF Tomography to device-free target tracking under a high-density distribution of wireless nodes, which will be further reviewed in Section 2.

To realize passive tracking with a low density of nodes, in this paper we focus on passive tracking schemes based on the geometric theorem (GT). In such schemes, the tracking problem is considered with a two-dimensional (2-D) geometric scenario, and then plane geometric theorems are applied to estimate a crossing point (CP) of the device-free target on the LOSLs. The 2-D geometric scenario contains wireless nodes as points, including the accessing points (APs) and monitor points (MPs), and the LOSLs, which are considered straight lines between the MPs and APs. In Reference [8], two typical GT schemes—the Least Mean-square-error (LM) and the Geometrical Formulation (GF) scheme—are introduced. The LM-based tracking scheme simply adopts the LOSL triggering sequence for CP estimation, while in the GF-based tracking scheme, a geometrical model for the estimation of CPs on the LOSLs is formulated according to an LOSL triggering sequence and the target’s distance travelled. However, the roughly known speed of the target is essential for the determination of distance travelled in the GF scheme, which is unpractical enough for a device-free target.

In this paper, we also propose a novel tracking scheme based on the geometric theorem named the Intercept Theorem (IT), which is also known as Thales’ theorem [12]. The IT-based CP estimation is realized by the ratio of the elapsed times between LOSL triggering timestamps when the parallel LOSLs are satisfied and this liberates the need for prior knowledge of the target’s speed. Moreover, we also present an approach named the equal-rate traverse (ERT) algorithm for the IT scheme to estimate CPs when the parallel topology is distorted by active MP positioning deviation. Following this, we focus on the property analysis and performance evaluation of these three GT-based tracking schemes; the LM, GF, and IT schemes.

In general, the contributions of this paper are briefly summarized as follows:

(1) We propose the IT-based CP estimation algorithm to liberate the known-speed limitation of the existing GF-based scheme; moreover, we carry out the ERT algorithm to enable the IT-based scheme for CP estimation under non-parallel wireless node distribution;
(2) The performance of GT-based tracking schemes, including the proposed IT-based scheme and the existing LM and GF schemes, is adequately evaluated in terms of MP position deviation, node density, and trajectory styles;
(3) Both the spatial and temporal characteristics of RF Tomography are explored by a mass of practical experiments to ensure RF Tomography detection.

The rest of this paper is organized as follows: Section 2 gives a comprehensive review of the work in the current passive tracking field; Section 3 investigates both spatial and temporal characteristics of RF Tomography to guarantee LOSL triggering detection; in Section 4, the IT scheme is proposed after two existing geometric theorem-based tracking schemes, including the LM scheme and GF scheme, are reviewed; for performance evaluation of the proposed IT scheme under assumed MP estimation deviation, the ERT algorithm is presented in Section 5; in Section 6, the properties of the three schemes are analyzed; meanwhile, their performance is evaluated through practical experiments under various trajectories and LOSL densities. Finally, conclusions and future directions are given in Section 7. In addition, for clarity, ‖X−Y‖ indicates the Euclidean distance between point X and Y, and $l_{x}\cap l_{y}$ presents the intersection of straight lines $l_{x}$ and $l_{y}$.

## 2. Related Works

The passive tracking method adopting radio frequency tomography has been recently addressed as an emerging issue in wireless peer-to-peer networks. A conventional approach is based on the offline-training and online-matching methodology. For an example, the extension system of Reference [1]—*Nuzzer*—was introduced in a Wi-Fi environment [13]. Two phases are disposed in this system: a radio map that stores information on the signal strength at different locations in the area of interest is constructed during an offline phase, while during the online phase, the *Nuzzer* system uses the signal strength samples instantaneously received from the APs at the MPs and compares them to the passive radio map to estimate the location of the tracked entity. Similar works are referred to in Reference [14]; for example, Viani et al. divided the domain of interest into square regions and used a Support Vector Machine to train the a priori relationship between the target’s location and RSS measurements. In the testing phase, the a posteriori probability is then used to locate the target in a region by fusing the RSS measurements of all LOSLs with the a priori relationship. Other, similar works that adopt such a two-phase approach can be found in Reference [9].

The above-mentioned offline-training and on-matching methods, however, suffer from the time-varying environment. Moreover, it is tedious work to update the offline database regularly. Therefore, Wilson et al. paid attention to the LOSLs, and employed the skew-Laplace distribution to model RSS attenuation for tracking a stationary target in a radio frequency WSN [15]. Instead of offline training, only a short calibration of RSS from a communication link is required for tracking both dynamic and static device-free targets. Nannuru et al. made a modification to the skew-Laplace measurement model to build a new magnitude measurement model, which was successfully adopted to realize multi-target tracking in indoor environments by particle-based algorithms [7].

The LOSL-based approach has received more and more attention. In Reference [16], the monitoring area in the WSNs is divided into grids, according to maps of the shadowed and non-shadowed links, and the feasible and infeasible grids are distinguished. Finally, the Bayesian grid approach is used to determine the device-free target positioning information. Another LOSL-based tracking scheme [17] is proposed by Wang et al. They utilized not only the observation information of the shadowed LOSLs, but also the prior information involved in the previous estimations and the constraint information involved in the non-shadowed links, which ensure its robust location estimation performance. The proposed approach also represents the prior information, observation, and posterior information with grid maps, and can be implemented with a series of simple grid multiplication and addition operations, which makes it a lightweight scheme suitable for hardware-limited application. The extension of these works is referred to in References [18,19].

However, because of the multipath effect, the LOSL shadowing is sometimes detected to be negative. Thus, some researchers pay attention to outlier LOSL rejection using probabilistic approaches, rather than improving the RF Tomography detection of the LOSLs. An example is presented in Reference [20], wherein Xiao et al. proposed a novel nonlinear optimization approach with outlier link rejection for RSS-based device-free localization which consists of three key strategies including: (1) affected link identification by differential RSS detection; (2) outlier link rejection via geometrical positional relationship among links, and (3) target location estimation by formulating and solving a nonlinear optimization problem. To mitigate the uncertainty of the LOSLs, instead of an RF network, Mao et al. presented a tracking system named “iLight” using optical sensor nodes and one base station in Reference [21]; they proposed probabilistic tracking strategies to track both single and multiple targets. The experimental results show that the iLight scheme is not only able to compute the moving trajectories of targets efficiently, but also to study the properties of moving targets (e.g., height).

As a matter of fact, a common shortcoming of the existing LOSL-based methods mentioned above is that they require high-density wireless nodes forming a dense network to ensure that all of the corners in the monitored area are covered with LOSLs, in order to guarantee reliable tracking accuracy, which thus decreases their practicability. In contrast, the GT-based passive tracking schemes discussed in this paper aim to provide another way to track device-free targets, that is, to find the CPs of the target on the LOSLs to indicate its trajectory in a discrete form. Instead of using a group of LOSLs for the locating process, the GT-based tracking schemes pay attention to individual LOSLs, which thus can efficiently decrease the equipment requirement for the high density of wireless nodes.

## 3. Characteristics of RF Tomography

**Figure 1:**
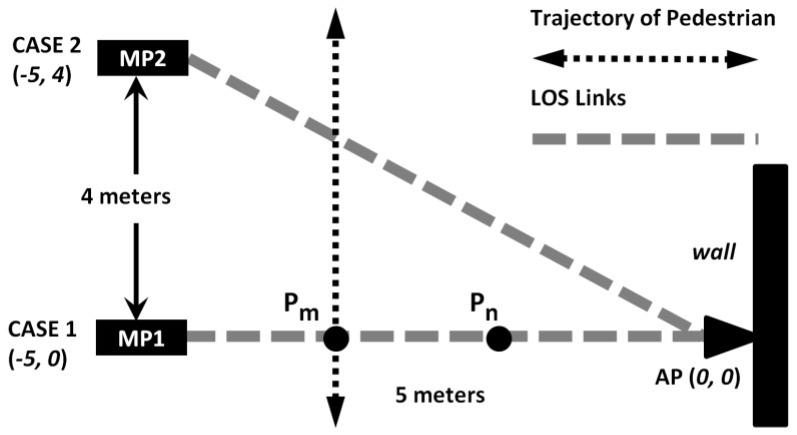
Two monitoring point (MP) locations to investigate temporal characteristic of Radio Frequency (RF) Tomography: Case 1: the MP is located at (−5, 0); Case 2: the MP is located at (−5, 4).

**Figure 2:**
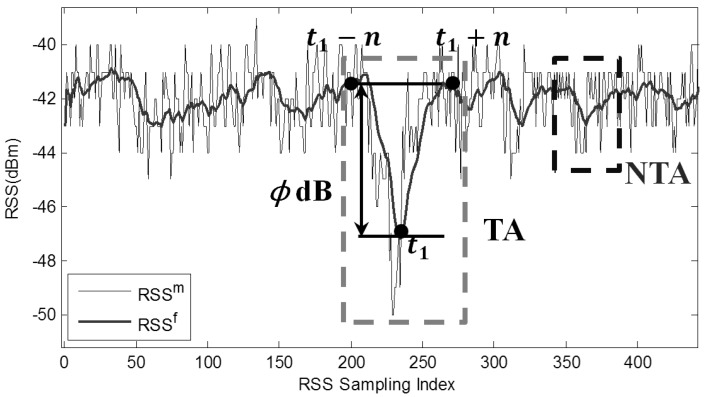
Received signal strength (RSS) measurement when a pedestrian passes through the line-of-sight (LOSL) at time index t.

To investigate RF tomography, as shown in Figure 1, one AP is attached to the wall and two MPs with known position information are used to collect RSS data from the AP. When a target passes through the LOSL between MP1 and AP at time index t, an RSS attenuation appears and this LOSL is triggered, as shown in Figure 2. Note that the Android-powered handhold devices SHW-M240Ss (Manufacturer: Samsung, Seoul, Korea) are adopted here as the MPs, while ZIO-AP1500N Routers (Manufacturer: Millennium Network Frontier, Suzhou, China) are used as the APs. The RSS collection system is operating at 2.4 GHz under a Wi-Fi communications environment, in which the APs send data to a specific receiver through a channel with a fixed frequency using a constant transmitting power, and thus the RSS measured at the receiver will be maintained at a relative stable value under a static environment [22].

As the characteristics of RF Tomography, including spatial and temporal characteristics, are foundational knowledge necessary for the implementation of GT-based passive tracking schemes, these are investigated beforehand in order to guarantee the LOSL triggering detection.

### 3.1. Spatial Characteristic

**Figure 3:**
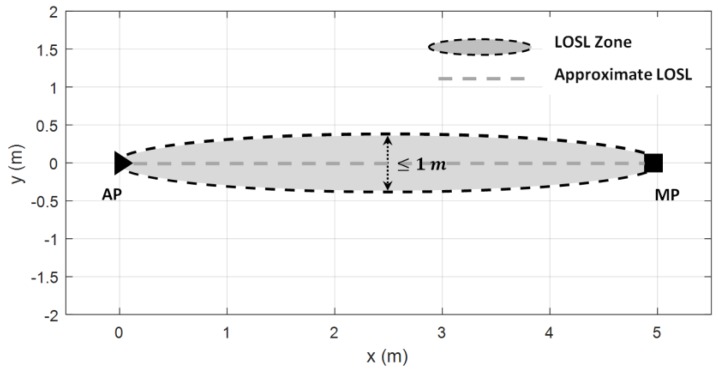
When the mean attenuation g(x) is larger than 3 dB, and ϕ and $\sigma_{\lambda }$ are set as 5 and 0.3, respectively, the theoretical LOSL zone width is smaller than 1 m (the practical experiment result is referred to in Reference [8]) and thus can be approximated as a straight line.

As presented in our previous work [8], when LOSL triggering is detected, we cannot directly determine the exact crossing point ($P_{n}$ or $P_{m}$, or other points on the LOSL in Figure 1) by only distinguishing the feature of RSS attenuation. This is further proven by Li’s pixel-free model in Reference [23]. In Li’s model, the relationship between the mean of attenuation g(x) and the position of the target, x, is expressed as follows:

(1) $$g\left(x\right)=\phi \cdot exp\left(-\frac{\lambda \left(x\right)}{\sigma_{\lambda }}\right)$$

where ϕ is the scalar weight parameter and $\sigma_{\lambda }$ is the parameter deciding the attenuation amplitude with respect to λ(x). Generally, ϕ ranges from 3 to 7 and $\sigma_{\lambda }$ ranges from 0.2 to 0.4, and they are both attenuation constants estimated by an online calibration process with respect to the physical properties of the targets and nodes from a mass of experiments. λ(x) denotes a measure of the distance from the target to the LOSL between MP and AP, and is defined as follows:

(2) $$\lambda \left(x\right)=d_{1}\left(x\right)+d_{2}\left(x\right)-d_{12}$$

where $d_{1}\left(x\right)$ and $d_{2}\left(x\right)$ are the distances between the target and the two ends of the LOSL, and $d_{12}$ is the length of the LOSL. According to this model, the LOSL zone is an elongated ellipse area (the two focal points of the ellipse are the AP and MP) when the mean of attenuation is given (see Figure 3); here, we term this area as the LOSL zone and approximate this LOSL zone as a straight line.

In model (1), when λ(x) equals 0—that is, when the target is located on the LOSL—the attenuation would be an invariant regardless of where target locates on the LOSL; this confirms our conclusion that it is not feasible to find the exact CP by RSS attenuation [8].

### 3.2. Temporal Characteristic

The speed of the target is an important issue for detecting RF Tomography. The maximum allowed speed of the target is directly related to the RSS sampling rate in the passive tracking schemes based on RF Tomography. Assuming that the target is moving at a fast speed and the RSS sampling rate is not high enough, we will fail to recognize the RF Tomography and thus the tracking work cannot be launched; that is, if the LOSL triggering is not detected, the CP determination on that LOSL will be unattainable. Therefore, to ensure LOSL triggering detection, we investigate the relationship between the speed of the target and the sampling rate of MP, as the RF Tomography temporal characteristic.

In this experiment, the AP is fixed at (0, 0) and two cases for MPs are designed as shown in Figure 1. These two cases present a preliminary calibration for the experiments setup in the following performance evaluation section, in which the longest LOSL is the same situation as that given in Case 2.

The RSS sampling rate is set as 10 Hz, and the two LOSLs in two cases are walked across along the dotted trajectory with different speeds (30 cm/s, 60 cm/s, and 90 cm/s) through random CPs. We adopted a sliding weighted mean (SWM) filter with a sliding window width W to smooth the RSS samples [1]:

(3) $${\mathrm{RSS}}_{t}^{f}=\sum_{i=0}^{W-1}C_{i}\cdot {\mathrm{RSS}}_{t-i}^{m}$$

where ${\mathrm{RSS}}_{t}^{m}$ is the measured raw RSS value at time index t. ${\mathrm{RSS}}_{t}^{f}$ represents the RSS value after filtering. $C_{i}$ is the weight, and it is assigned by the ruler $C_{i}=2\left(W-i\right)/W\left(W+1\right)$, which means that the currently measured RSS values are assigned with a larger weight; in this way, we can mitigate the phase shift problem and improve the real-time performance of the system. The RSS information is presented in Figure 2, when an LOSL is triggered at time index $t_{1}$. Here, we define that an attenuation action with amplitude ϕ dB is measured at the time index t, if the filtered RSS value at the time index t is the bottom of a valley with ϕ dB depth, which can be mathematically denoted by:

(4a) $$\left|{\mathrm{RSS}}_{t}^{f}-{\mathrm{RSS}}_{t-n}^{f}\right|\geq \phi \;\&\;\left|{\mathrm{RSS}}_{t}^{f}-{\mathrm{RSS}}_{t+n}^{f}\right|\geq \phi$$

(4b) $$\mathrm{and}\;\left({\mathrm{RSS}}_{t}^{f}-{\mathrm{RSS}}_{t-n}^{f}\right)\cdot \left({\mathrm{RSS}}_{t}^{f}-{\mathrm{RSS}}_{t+n}^{f}\right)<0.$$

when a device-free target is walking around a monitored area, two kinds of attenuation actions might be detected and classified as a traverse attenuation (TA) or a non-traverse attenuation (NTA) according to whether the target passed by the LOSL or not, as depicted in Figure 2. An NTA is caused by environment noise while the target is walking around without LOSL traverse. A safe threshold—$\phi^{\prime }$—for distinguishing the TA from the NTA, should be set larger than most NTA amplitudes to avoid false positive TA detection and should be smaller than most TA amplitudes to avoid missing negative TA detection errors. An optimal window width W of the SWM filter should be selected to mitigate NTA while guaranteeing TA amplitude.

**Figure 4:**
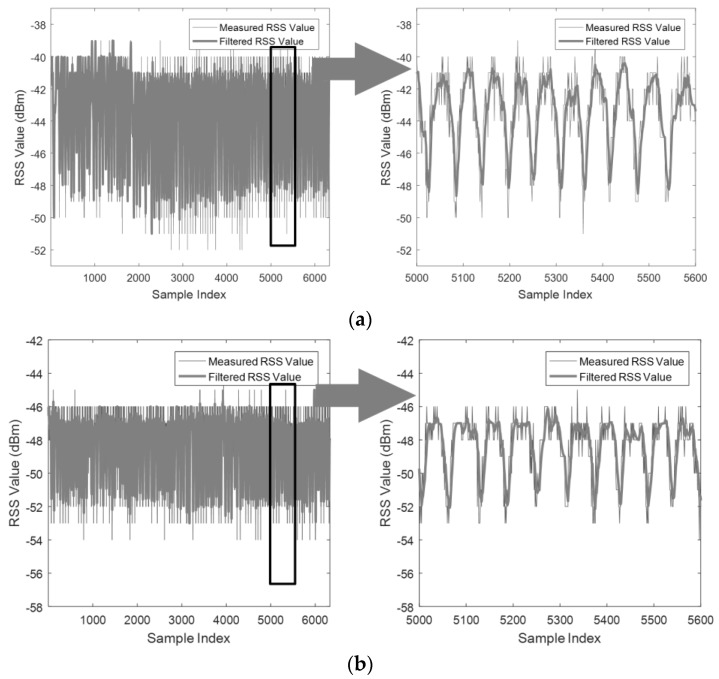
The RSS measurement when a pedestrian periodically walks across the LOSLs at a speed of 30 cm/s in (**a**) Case 1 and (**b**) Case 2.

**Figure 5:**
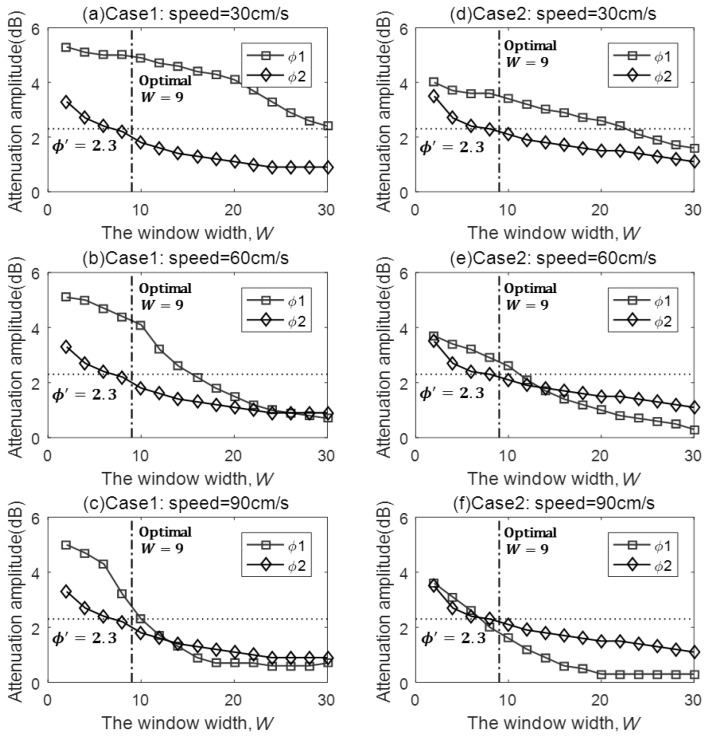
The attenuation amplitudes ϕ1 and ϕ2 under various speeds in the two cases.

To obtain the optimal W and the safe threshold $\phi^{\prime }$, the statistical method is performed as follows. We conducted the LOSL traverse about 500 times at each speed for both cases (a portion of measured RSS is given in Figure 4) and tallied the cumulative distribution function (CDF) of the TA amplitude, $F_{\mathrm{TA}}\left(\phi \right)$. Meanwhile, the CDF of the NTA amplitude, $F_{\mathrm{NTA}}\left(\phi \right)$, was obtained from the RSS measurement under the scenario that the target is randomly walking around the LOSLs without triggering the LOSLs, that is, keeping away from the LOSL zone. In this way, we established a more reasonable CDF of NTA amplitude to mitigate the false positive LOSL triggering caused not only by the hardware and propagation noise but also by the RSS fluctuation from the moving target. Then, we determined both the smallest TA amplitude ϕ1 when $F_{\mathrm{TA}}\left(\phi 1\right)=1\%$ and the largest NTA amplitude ϕ2 when $F_{\mathrm{NTA}}\left(\phi 2\right)=99\%$, which are illustrated in Figure 5. In essence, in the promise of $\phi 1>\phi^{\prime }>\phi 2$, the false positive rate of TA detection will be smaller than 1% since $1-F_{\mathrm{NTA}}\left(\phi 2\right)<1\text{\%}$. In the same way, the missing negative rate is likewise restrained below 1% since $F_{\mathrm{TA}}\left(\phi 1\right)<1\text{\%}$. Thus, an appropriate threshold, which is smaller than ϕ1 and larger than ϕ2, should be carefully selected in terms of W. According to the statistical results in Figure 5, the optimal W can be set at around 9, shown by the vertical dash-dot line, and the threshold $\phi^{\prime }$ can be set around 2.3 dB, shown by the transverse dotted line representing the best interest over all six scenarios.

The maximum allowed speed of the target was more than 90 cm/s in Case 1 and around 60 cm/s in Case 2, as shown in Figure 5c,e. It is true that the higher RSS sampling frequency will enlarge the maximum allowed speed; however, this will lead to energy dissipation. For example, the RSS sampling rate in topical smart terminals is limited to a maximum of 1 Hz in common Wi-Fi scenarios, for the consideration of energy conservation [24].

Theoretically, the size of the sliding window should be increased with a higher sampling rate. This is because when more RSS values are measured per second, the larger filtering window can better stabilize the RSS fluctuation when the target is not traversing the LOSLs, that is, decreasing the amplitude of the NTA, while enough RSS measurements can still ensure the amplitude of the TA when the target is traversing an LOSLs. On the contrary, a smaller filtering window size should be chosen when the RSS sampling rate is not sufficiently large.

The above discussion intends to provide a strategy for guaranteeing LOSL triggering detection in a specific wireless environment without the loss of generality, and can be considered as a preliminary calibration phase for the subsequent experiment section. In the following, we assume that all the TAs can be accurately detected once the target walks through the LOSLs and the LOSL triggering sequence can be accurately measured, relying on the prior knowledge mentioned above.

## 4. GT-Based Passive Tracking Schemes

**Figure 6:**
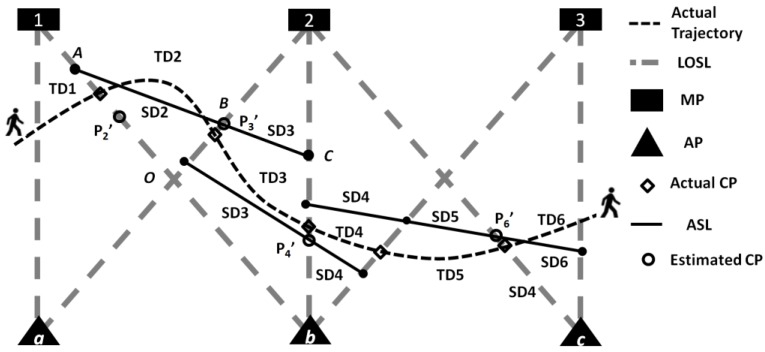
A common abstracted two-dimensional (2-D) passive tracking scenario under low density wireless nodes.

The reference nodes APs and MPs as well as the LOSLs between them form a 2-D geometric topology covering the monitoring area, as shown in Figure 6. To estimate the CPs on the LOSLs of the dynamic target and reveal its moving trajectory information, geometric theorems are adopted, which is termed GT-based CP estimation. In this section, a novel CP estimation scheme exploiting the intercept theorem is presented. Prior to that, we review the existing CP estimation schemes, including Least Mean-square-error and Geometric Formulation. It should be noted that the tracking work presented in this article is for a single target; although GT-based passive tracking schemes have the ability to track multiple targets, multiple targets tracking work is beyond the scope of this paper.

In Figure 6, three AP-MP pairs form an LOSL web, monitoring a 5×8 m^2^ area (the uniform space of AP-MP pairs is set as 4 m). We set APa as the origin [0, 0], APb and APc are located at [0, 4] and [0, 8], while MP1, MP2, and MP3 are located at [5, 0], [5, 4], and [5, 8], respectively. When the device-free target passes by the monitoring area, the seven LOSL triggering sequences, LOSL(i),  i=1, 2,…,7, are successively measured as $l_{a,1}$, $l_{b,1}$, $l_{a,2}$, $l_{b,2}$,$\;l_{b,3}$, $l_{c,2}$, and $l_{c,3}$, noting that $l_{X,Y}$ denotes the LOSL between APX and MPY. The elapsed times between LOSL(i) and LOSL(i+1) triggering are recorded as T(i),i=1,2,…,6.

The formula of the straight line LOSL(i) can be expressed in a slope-intercepted form as $y=k_{i}\times x+b_{i}$, where $k_{i}$ and $b_{i}$ are known information once the reference nodes are fixed. For example, the formula of LOSL(2), that is, $l_{b,1}$ is calculated as y=1.25 x+4 and the $l_{b,2}$ can be approximated as a straight line with huge slope, such as $y=10^{3}\;x-4\times 10^{3}$.

### 4.1. Least Mean-Square-Error Algorithm

When the target passes by an LOSL, and a TA on this LOSL is detected, the CP could be any point on that LOSL according to the spatial characteristic of RF Tomography discussed previously. We empirically prefer to decide the middle point of this LOSL as the CP to minimize the mean-square-error of CP estimation. Nevertheless, if the LOSL triggering sequence is considered, we can narrow the possible CP area to the restricted segment on the LOSL.

For example, in Figure 6, when we want to estimate the CP on LOSL(2), while LOSL(1) is $l_{a,1}$, it is impossible that the CP is located at the segment Ob, because after triggering $l_{a,1}$ the target cannot pass by segment Ob without triggering $l_{a,2}$. Thus, we set the middle point of segment O1 as the estimated CP. Similarly, if we want to estimate the CP on LOSL(3), after determining that the previous CP is on O1, its more reasonable that the target would be passing by middle point of the segment O2 on LOSL(3). This method is called the LM scheme. A brief description of the process of LM-based CP estimation is presented in Algorithm 1.

**Algorithm 1: CP Estimation on**
LOSL(i)
**based on the Least-square-error Method.****Initialization:** Obtain the coordinates of all wireless sensor nodes, as well as the topology information of all LOSLs, that is, their formulas.**Step** **1.**Launch the algorithm when LOSL(i) is triggered;**Step** **2.**List all the segments on LOSL(i) and obtain the coordinate set of each segment;**Step** **3.**Find the unique possible segment where the CP may be located by using the LOSL triggering sequence;**Step** **4.**Set the middle point of the unique possible segment as the CP.

### 4.2. Geometric Formulation Algorithm

If the speed of the device-free target is roughly known as $v_{knw}$, the distance travelled during T(i) can be known as $TD\left(i\right)=v_{knw}\cdot T\left(i\right)$. The GF-based CP estimation algorithm [8] utilizes TD(i) and LOSL(i) as measurements for CP estimation. The estimation process of the third CP, $P_{3}$, on LOSL(3) in Figure 6 is taken as an example to describe the GF-based CP estimation.

Firstly, we approximate that the trajectory over TD(2) and TD(3) is a straight line called the approximate straight line (ASL), because the ASL is passing the CP on LOSL(3), presented as $P_{3}=\left[x_{3},\;y_{3}\right]$, so the ASL formula in point-slope form is denoted by:

(5) $$y-y_{3}={\hat{k}}_{3}\;\left(x-x_{3}\right)$$

where ${\hat{k}}_{3}$ is the slope of the ASL. $x_{3}$, $y_{3}$, and ${\hat{k}}_{3}$ are all unknown values. Because $P_{3}$ is located on LOSL(3), so we have $y_{3}=k_{3}\;x_{3}+b_{3}$; thus, Equation (5) can be rewritten as:

(6) $$y={\hat{k}}_{3}\;x+{\hat{b}}_{3}$$

where ${\hat{b}}_{3}=\left(k_{3}-{\hat{k}}_{3}\right)\;x_{3}+b_{3}$. In this way, the unknown variables in the formula of the ASL are $x_{3}$ and ${\hat{k}}_{3}$.

Secondly, the ASL is intersected with LOSL(2), LOSL(3), and LOSL(4) at points A, B, C and their coordinates $A={\left[x_{A},y_{A}\right]}^{T}$, $B={\left[x_{B},y_{B}\right]}^{T}$, $C={\left[x_{C},y_{C}\right]}^{T}$ can be calculated by:

(7a) $$\left[\begin{array}{c} x_{A}\\ y_{A} \end{array}\right]={\left[\begin{array}{cc} -{\hat{k}}_{3} & 1\\ -k_{2} & 1 \end{array}\right]}^{-1}\cdot \left[\begin{array}{c} {\hat{b}}_{3}\\ b_{2} \end{array}\right]$$

(7b) $$\left[\begin{array}{c} x_{B}\\ y_{B} \end{array}\right]=\left[\begin{array}{c} x_{3}\\ k_{3}\;x_{3}+b_{3} \end{array}\right]$$

(7c) $$\left[\begin{array}{c} x_{c}\\ y_{c} \end{array}\right]={\left[\begin{array}{cc} -{\hat{k}}_{3} & 1\\ -k_{4} & 1 \end{array}\right]}^{-1}\cdot \left[\begin{array}{c} {\hat{b}}_{3}\\ b_{4} \end{array}\right]$$

The lengths of two segments AB and BC are calculated as the Euclidean distances SD(2) and SD(3), respectively:

(8) SD(2)=‖A−B‖, SD(3)=‖B−C‖

Hence, there would be one unique solution of ${\hat{k}}_{3}$ and $x_{3}$ deciding the best-fitting ASL, whose SD fit TD best. To minimize the mean-square-error between segment lengths and the distances travelled, the arguments can be found to maximize a nonlinear function, which is given as follows:

(9) $$\arg\;\underset{x_{3},\;{\hat{k}}_{3}}{\max}\;1/\sum_{i=2}^{3}{\left(TD\left(i\right)-SD\left(i\right)\right)}^{2}$$

Finally, using optimization algorithm [25], the optimal value of the two arguments $x_{3},\;{\hat{k}}_{3}$ in the above model can be determined, after which the estimated CP is obtained by putting the optimal value of $x_{3}$ into the known LOSL(3) formula. Generally, the problem of locating the CP is formulated as a geometric model trying to find the best-fitting ASL, and the CP is estimated as the intersection of the best-fitting ASL and the corresponding LOSL. A brief description of the process of the GF-based CP estimation is presented in Algorithm 2.

**Algorithm 2: CP Estimation on**
LOSL(i)
**based on Geometric Formulation.****Initialization:** Obtain the coordinates of all wireless sensor nodes, as well as the topology information of all LOSLs, that is, their formulas; roughly know the pedestrian speed $v_{knw}$;**Step** **1.**Launch the algorithm when LOSL(i+1) is triggered;**Step** **2.**Find the unique possible segment where the CP may be located as the restricted region;**Step** **3.**Build the matching model in Equation (9) between the ASL and measured distances travelled $v_{knw}\cdot T\left(i\right)$ and $v_{knw}\cdot T\left(i+1\right)$;**Step** **4.**Optimize the model (9) to determine the best-fitting ASL;**Step** **5.**Obtain the intersection of the best-fitting ASL and LOSL(i) as the estimated CP.

### 4.3. Intercept Theorem Algorithm

In the GF scheme, it is assumed that the speed of the target is known. Even though the speed of a human target can be roughly estimated as a person’s normal walking speed—assumed to be 0.7 m/s—this constant is not necessarily accurate enough, and its performance would deteriorate when the target is not walking at the normal speed. Hence, another GT-based tracking scheme adopting the Intercept Theorem is here proposed to estimate CPs without the need for the known speed of the target.

In plane geometry, suppose that a point S is fixed in the region between a pair of parallel lines, and another straight line MN, which is not parallel with the parallel line pair, passes through this point S in addition to intersecting the parallel line pair at points M and N, respectively. According to the Intercept Theorem in 2-D geometry [17], no matter how the line AB passes through the point S at any angle, the ratio ‖M−S‖:‖S−N‖ will always be a constant, R. For example, when another straight line $N^{\prime }M^{\prime }$ passes through the point S at a different angle and intersects the parallel straight line pair at points $M^{\prime }$ and $N^{\prime },$ we will have: $\|M-S\|:\|S-N\|=\|M^{\prime }-S\|:\|S-N^{\prime }\|=R$. With this principle, we can launch the IT-based CP estimation.

What can be measured from the WSN scenario in Figure 6 are the elapsed times between LOSL triggering points, while the speed of the device-free target is unknown. When the LOSL density is high to a certain extent, two reasonable assumptions can be drawn from the GF-based algorithm. First, it is a reasonable assumption that the target is moving at a constant speed $v_{unk}$ during contiguous T(i)s; second, it is assumed that we can also approximate the trajectory over a pair of parallel LOSLs as a straight line, called the ASL.

Under the above two assumptions, when we want to estimate the CP on LOSL(i), and LOSL(i−1) and LOSL(i+1) are a parallel pair, it is determined that LOSL(i) intersects with LOSL(i−1) and LOSL(i+1) at points $M={\left[x_{M},y_{M}\right]}^{T}$ and $N={\left[x_{N},y_{N}\right]}^{T}$, which can be expressed by:

(10a) $$\left[\begin{array}{c} x_{M}\\ y_{M} \end{array}\right]={\left[\begin{array}{cc} -k_{i} & 1\\ -k_{i-1} & 1 \end{array}\right]}^{-1}\cdot \left[\begin{array}{c} b_{i}\\ b_{i-1} \end{array}\right]$$

(10b) $$\left[\begin{array}{c} x_{N}\\ y_{N} \end{array}\right]={\left[\begin{array}{cc} -k_{i} & 1\\ -k_{i+1} & 1 \end{array}\right]}^{-1}\cdot \left[\begin{array}{c} b_{i}\\ b_{i+1} \end{array}\right]$$

The unknown CP, $P_{i}$, and known intersections M, N are all located on LOSL(i). Thus, we suppose that the length ratio of the segment $MP_{i}$ and $NP_{i}$ is R(i):

(11) $$\|P_{i}-M\|/\|P_{i}-N\|=R\left(i\right)$$

Meanwhile, the ASL is cut by LOSL(i−1), LOSL(i), and LOSL(i+1) into two segments: SD(i−1) and SD(i). Because we approximate the ASL as the trajectory over a pair of parallel LOSLs, the segments are surely proportional to the actual time travelled: TD(i−1)∝T(i−1) and TD(i)∝T(i). Hence, we can calculate the ratio R(i) by:

(12) R(i)=SD(i−1)/SD(i)≅TD(i−1)/TD(i)

where R(i) is the ratio T(i−1)/T(i) when the speed $v_{unk}$ is divided out. Generally, points M and N are MPs or APs. Then $P_{i}$ is determined by putting R(i) into Equation (11).

To specifically describe this algorithm, we employ the estimation procedure of crossing point $P_{4}$ on LOSL(4), that is, $l_{b,2}$, in Figure 6 as an example. As for crossing point $P_{4}$ on $l_{b,2}$, the previously and subsequently triggering LOSLs are $l_{a,2}$ and $l_{b,3}$, respectively. These two LOSLs make a parallel straight line pair; thus, the ratio is determined by: R(4)=T(3):T(4). The points M and N are obviously MPb and AP2, hence, according to $\|{P_{4}}^{\prime }-MP2\|/\|{P_{4}}^{\prime }-APc\|=R\left(4\right)$, we set the point ${P_{4}}^{\prime }$ as the estimated CP:

(13) $${P_{4}}^{\prime }=\frac{\mathrm{AP}b+\mathrm{MP}2\cdot R\left(4\right)}{1+R\left(4\right)}$$

In addition, for the general case where the nearby LOSL(i−1) and LOSL(i+1) are not the parallel pair anymore, the IT-based CP estimation algorithm is presented in Algorithm 1. For the CP on LOSL(6), ${P_{6}}^{\prime }$, the adjacent parallel pair consists of LOSL(7) and LOSL(5), which are not a parallel straight line pair. Then, according to Algorithm 1, the nearest parallel straight line pair consists of LOSL(4) and LOSL(7), therefore the ratio of LOSL(6) is:

(14) $$R\left(6\right)=\sum_{j=4}^{5}T\left(j\right)/T\left(6\right)$$

Thus, ${P_{6}}^{\prime }$ can be determined by:

(15) $${P_{6}}^{\prime }=\frac{\mathrm{AP}c+MP2\cdot R\left(6\right)}{1+R\left(6\right)}$$

since intersections LOSL(6)∩LOSL(4) and LOSL(6)∩LOSL(7) are MP2 and APc, respectively. In general cases, the IT-based scheme can be presented as Algorithm 3.

**Algorithm 3: CP Estimation on**
LOSL(i)
**based on the Intercept Theorem.****Initialization:** Obtain the coordinates of all wireless sensor nodes, as well as the topology information of all LOSLs, that is, their formulas.**Step** **1.**Launch the algorithm when LOSL(i+1) is triggered;**Step** **2.**Successively check whether LOSL(i−n) is parallel with LOSL(i+1) in the reverse order, n∈[1, 2, ⋯,i−1];n=1;**while** (1)  **if** LOSL(i−n) is parallel to LOSL(i+1)    Obtain the elapsed time ratio R(i) according to Equation (14):     $R\left(i\right)=\sum_{j=1}^{n}T\left(i-j\right)/T\left(i\right);$  **else**    Check next LOSL by: n=n+1;  **end****end****Step** **3.**Retrieve the formula of LOSL(i+1), LOSL(i), LOSL(i−n), and obtain the intersections:$M,\;\left(x_{M},y_{M}\right)$ is the intersection of LOSL(i+1) and LOSL(i);$N,\;\left(x_{N},y_{N}\right)$ is the intersection of LOSL(i−n) and LOSL(i)**Step** **4.**Estimate the CP according to Equation (15):P=(N+MR)/(1+R).

### 4.4. Error Analysis of GT-Based CP Estimation Schemes

Generally, when the MPs and APs are distributed with exact known positions, the error can result from two aspects: the theoretical error and the measurement error.

The theoretical error originates from the algorithm design. Essentially, we roughly determine the CP as the middle point of the restricted segment on the triggering LOSL, and this results in the theoretical error in the LM scheme. Similarly, we approximate the travelled displacements between the LOSLs as straight lines in the GF and IT schemes, which is sometimes incorrect; the target may walk in a curved line or turn around. Such actions will damage this approximation and lead to theoretical error in GF-based and IT-based schemes.

The triggering elapsed times—T(i)—is the source of the measurement error. Because of the environment noise and practical systematic deviation, the LOSL triggering timestamps are measured with the deviation, and this deviation apparently has negative effects on the CP estimation accuracy.

## 5. CP Estimation under MP Positioning Deviation

The CP estimation methods mentioned above are conducted for scenarios in which the MPs and APs are predetermined as a known constant topology. In this section, we introduce the approach of CP estimation when the MPs’ position is no longer considered as an explicitly known value. Generally, in dynamic WSN scenes, the APs are always fixed at known coordinates (e.g., sink nodes) while the MPs are sometimes free mobile nodes (e.g., handheld terminals). To estimate the CPs of a device-free target on LOSLs between MPs and APs in such dynamic scenes, active positioning should first be performed to establish the MPs’ instant coordinates. Plenty of research studies have focused on the active positioning field; therefore, here we will not present the active positioning algorithms for locating MPs but will rather discuss how to estimate CPs when the MP position is established with deviation.

**Figure 7:**
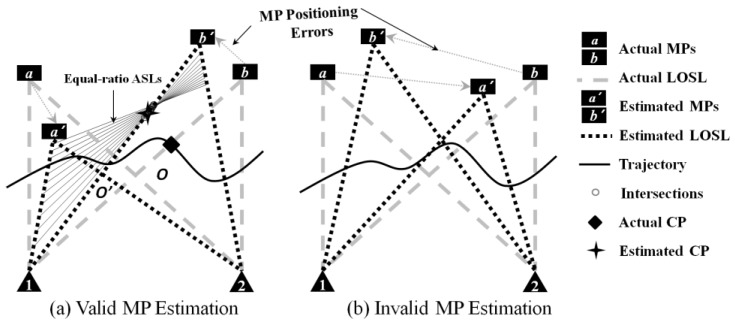
The crossing point (CP) estimation based on the equal-ratio transverse (ERT) method under noisy MP estimation.

According to the actual MP deployment of Figure 7, if the device-free target is passing though the monitoring area from left to right, and the LOSL triggering sequence LOSL(i), i=1, 2,…, 4, is measured as $l_{a,1}$ $l_{a,2}$, $l_{b,1}$, and $l_{b,2}$. When the MPa and MPb positions estimated from the active positioning techniques deviate from the ground truth to points MP$a^{\prime }$ and MP$b^{\prime }$, two kinds of shifted topologies may occur. For the first valid topology (see Figure 7a), the measured sequence is still logical for the CP estimation, although the topology is distorted. However, when MPs’ positions are estimated as the second topology (see Figure 7b), it would be illogical for the target to have triggered $l_{a,2}$ and $l_{b,1}$ successively since $l_{a,2}$ and $l_{b,1}$ are not neighboring LOSLs in the estimated topology. We would therefore discard the illogical estimated topology caused by invalid MP estimation, and only launch CP estimation on the estimated LOSLs when a valid MP estimation is achieved. A valid MP estimation means that the estimated LOSL topology conforms to the measured triggering sequence.

It is known from the previous section that LM and GF schemes have no need for parallel LOSLs; that is, they can be easily implemented once a valid LOSL topology is established. As for IT-based CP estimation, which requires parallel LOSLs, the instantaneously estimated LOSLs may not always be distributed in parallel to each other. To estimate CPs using the IT algorithm under such non-parallel situation, we here propose an approach named the equal-ratio traverse (ERT) method. Taking the CP estimation on LOSL(3), $l_{b^{\prime },1}$ for an example, the ERT method is described as follows.

Firstly, we also suppose that the target has walked in a straight line (denoted as the ASL). Utilizing the LOSL triggering sequence, we establish the following three facts:

(1) The ASL must intersect with the restricted segment inferred in the LM scheme. Specific to the CP estimation on $l_{b^{\prime },1}$, the ASL has to pass segment $O^{\prime }b^{\prime }$.
(2) The ASL must have intersections in the monitored area with all of the LOSLs inside the parallel pair, that is, the ASL must intersect segments $a^{\prime }1$ and $b^{\prime }2$.
(3) The ASL’s slope must be restricted to a certain range which is determined according to the topology. For the ASL of $l_{b^{\prime },1}$ its slope $k_{ASL}$ is limited to:
  (16) $$\theta_{ASL}=\arctan k_{ASL}\in \left(\theta_{a^{\prime },2},\theta_{b^{\prime },2}\right)$$
  where $\theta_{2,a^{\prime }}=\arctan k_{a^{\prime },2}$ and $\theta_{b^{\prime },2}=\arctan k_{b^{\prime },2}$ are the incline angles of $l_{a^{\prime },2}$ and $l_{b^{\prime },2}$, respectively.

Then, we uniformly divide the angle range in Equation (15) into N slots:

(17) $$\theta_{n}=\theta_{2,a^{\prime }}+\frac{n\cdot \left(\theta_{b^{\prime },2}-\theta_{a^{\prime },2}\right)}{N},\;n=1,\;2,\ldots ,\;N-1$$

Using Algorithm 1, we decide that the ratio R(3) on LOSL(3) is $\sum_{j=1}^{2}T\left(j\right)/T\left(3\right)$, because the adjacent parallel LOSL pair is composed of LOSL(1), $l_{a^{\prime },1}$ and LOSL(4), $l_{b^{\prime },2}$. This indicates that the ratio of the lengths of two segments on the ASL cut by $l_{a^{\prime },1}\;\&\;l_{b^{\prime },1}$ and $l_{b^{\prime },1}\;\&\;l_{b^{\prime },2}$ equals R(3).

The ASL satisfying this ratio and with the slope $\tan\left(\theta_{n}\right)$ being unique and recorded as the n-th equal-ratio ASL, and can be denoted in the slope-intercept form: $y={\hat{k}}_{n}x+{\hat{b}}_{n}$, where ${\hat{k}}_{n}=\tan\left(\theta_{n}\right)$ and ${\hat{b}}_{n}$ are determined by the formula of $l_{a^{\prime },1}$, $l_{b^{\prime },1}$, and $l_{b^{\prime },2}$, as well as the ratio R(3).

Finally, we traverse all of the equal-ratio ASLs, and then discard those that cannot satisfy the requirements presented in Equations (1) and (2). The intersections of $l_{b^{\prime },1}$ and the remaining equal-ratio ASLs are distributed on segment $O^{\prime }b^{\prime }\;$(marked by circles in Figure 7). Then, the centroid of these intersections will be taken as the estimated CP under noisy MP estimation.

It should be noted that we alternatively select the approximate parallel LOSL pairs, although the two LOSLs have no intersection in the monitoring area (here the approximate parallel LOSL pair is composed of $l_{a^{\prime },1}$ and $l_{b^{\prime },1}$). In the other words, when no parallel LOSL pair exists, the ERT method is presented as an expedient method for finding the CPs on LOSLs.

## 6. Property Analysis and Performance Evaluation

In this section, the passive tracking schemes based on geometric theorems including the LM scheme, GF scheme, and GT scheme, are evaluated in two respects. Firstly, their properties are analyzed to reveal their practicability in different usage scenarios. Secondly, the tracking accuracy is evaluated with practical experiments as functions of the node density, trajectory style, and MP position deviation.

### 6.1. The Property Analysis of the Passive Tracking Schemes

First of all, one prime advantage of the GT-based tracking scheme is the low-density requirement of wireless nodes compared to that of other existing RF Tomography-based passive tracking algorithms. As can be seen from Table 1, in similar scenarios with passive tracking accuracy under 1 m, the GT schemes need only half as much or even less node density than the other RF Tomography-based passive tracking schemes, which undoubtedly reduces the hardware requirement and increases the feasibility of the GT-based tracking schemes. In essence, most existing tracking schemes estimate the instant position of the device-free target only when the target is located at the intersecting region of a group of LOSLs; thus, they have the requirement of high LOSL density. For the GT-based tracking schemes, however, we pay attention to the individual LOSL triggering sequence and timestamps to estimate the CPs.

As for the three GT schemes, the analysis of their properties is necessary to determine their applicable situations. As shown in Table 2, the analysis is provided with four aspects. The first one is whether the speed of device-free target is required to be known as prior information. Except for the LOSL triggering sequence, the LM scheme needs no more information. The IT scheme can be implemented by information of the LOSL triggering sequence and the elapsed-time T(i), as the speed is divided out as a common divisor in Equation (12). The information on speed is necessary for the GF scheme to build a geometric model. Meanwhile, if we roughly set a speed for the device-free target, the GF scheme still works, although the tracking performance will be deteriorated.

The second issue is whether or not the LOSLs in the WSN need to be distributed parallel to one another. For a facilitating illustration, LM- and GF-based schemes are also presented in Figure 6, in which parallel LOSLs pairs are distributed. However, these two schemes are free from the wireless nodes distribution restriction, which was clearly declared in our previous work [8]. Nevertheless, in the IT-based scheme, parallel LOSLs pairs are necessary to apply the IT scheme to estimate CPs.

Although we proposed the ERT method for the IT-based CP estimation in cases where the LOSLs are not parallel pairs on account of the MP position deviation, it is apparent that the non-parallel LOSLs will surely deteriorate the CP estimation accuracy since the ERT method is merely an expedient for the non-parallel situation, as discussed above.

The third issue is the real-time performance of the tracking schemes. The LM scheme is a real-time tracking scheme since the CP, $P_{i}$, on LOSL(i) can be decided once LOSL(i) is triggered and before LOSL(i+1) is triggered. However, both GF and IT schemes are not exact real-time measurements. As illustrated in Section 4.2 and Section 4.3, $P_{i}$ on LOSL(i) is estimated by using LOSL(i+1) information; that is, only when the target passes by LOSL(i+1) can the system estimate the CP on LOSL(i).

The last issue is the computing cost. In general, the computing costs of the passive tracking schemes of geometric theorems are all rather low, since the tracking problem is abstracted into a simple 2-D geometric scenario. For a comparison among them, the GF scheme has a higher computing cost requirement than the other two schemes, because the geometric model building and model optimization algorithm are relatively complex. However, it is worth noting that the CP is also decided by several equations in the other two schemes.

### 6.2. The CP Estimation Performance versus the Trajectory Sinuosity and Wireless Node Density

**Figure 8:**
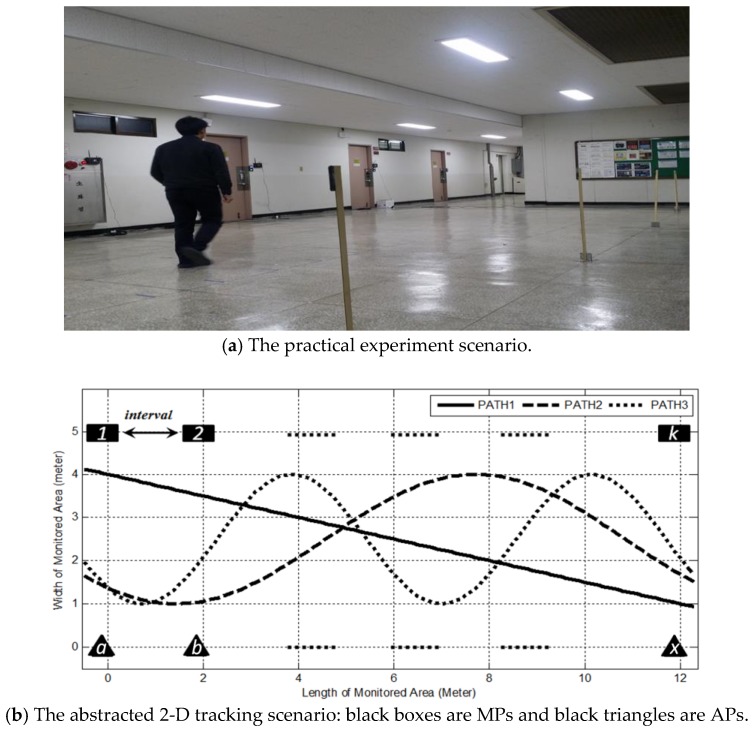
The experimental sketch to explore the effects of AP-MP density and trajectory sinuosity on the GF tracking scheme.

**Figure 9:**
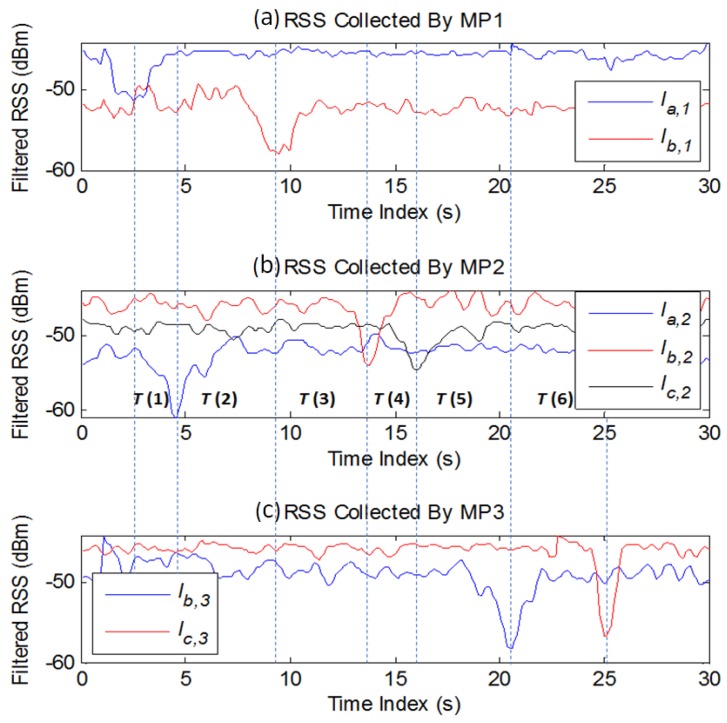
The measured RSS after filtering when three AP-MP pairs are distributed across the monitoring area (MP interval = 6 m).

To evaluate the performance of tracking schemes based on geometric theorems under different trajectories and LOSL densities, the experiment is deployed in a 5×12 m^2^ area (as shown in Figure 8). The experimental hardware consists of SHW-M240Ss as MPs and ZIO-AP1500N Routers as APs. During the tracking process, the MPs collect the RSS values from their nearby APs and send them to a central server in real time with a frequency of 10 Hz. The adopted RF Tomography detection parameters are the optimal parameters obtained in Section 2; the attenuation threshold $\phi^{\prime }$ is set as 2.3 dB and the SWM filtering window width W is predetermined as 9. After filtering and distinguishing the attenuation actions, the triggered sequence of LOSLs and elapsed times T(i) are recorded by the server (a preferred measurement is illustrated in Figure 9).

For simplicity, the LOSLs between two non-adjacent node pairs, such as $l_{c,1}$ or $l_{a,3}$ shown in Figure 8, are not counted in; this is also because the rather long LOSL distance of the AP-MP pair cannot guarantee triggering detection, as shown in Case 2 of Figure 4, since the 5×4 m^2^ structure seems to present a limitation for RF Tomography detection under a speed of 60 cm/s.

In addition, the LM scheme and the GF scheme have no requirement for LOSL parallel distribution; to evaluate all three schemes at the same test bed, the MP and the AP are placed in rectangular distribution. The distance from MP to AP is set as 5 m. The interval between adjacent MPs is changed in order to present different LOSL densities; decreasing the MP interval means increasing LOSL density, for instance, setting the MP interval as 3 m means there are five AP-MP pairs, that is, 10 nodes are uniformly placed in the monitoring area. The target moves along three paths with different trajectories from low sinuosity to high sinuosity marked as: PATH1, PATH2, and PATH3, at a speed of 60 cm/s to ensure LOSL triggering detection, as mentioned in Section 3.2.

**Figure 10:**
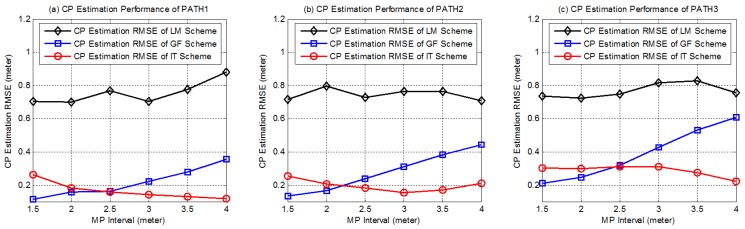
The CP estimation accuracy of the three geometric theorem-based tracking schemes with respect to the LOSL densities when the device-free target walks in three routes with different levels of sinuosity.

After 100 independent practical experiments, the root mean square errors (RMSEs) of CP estimation under various MP intervals for each path are shown in Figure 10. As shown in the figure, the performance of the GF scheme, which is mostly lower than 0.5 m, and that of the IT scheme, which is lower than 0.4 m, significantly outperform that of the LM scheme, which is around 0.8 m. This is reasonable because the LM scheme does not utilize any other information except for the LOSL triggering sequence. Inversely, although the accuracy is not good enough, the LM scheme is free from many limitations and more practical than the other two schemes.

It should be noted that, under the same MP interval, the path with higher sinuosity (more turning points) causes higher CP estimation RMSE for the IT scheme and the GF scheme. During the CP estimation processes in the IT and GF schemes, we approximate the trajectory in the adjacent elapsed time as a straight line; however, the more tortuously the target moves, the less reliable the approximation will be, and this leads to greater theoretical error. As for the LM-based CP estimation, in which no straight-line approximation is involved, the performance of the scheme does not depend on the trajectory tortuosity. Increasing LOSL density can make the elapsed time T(i) between two LOSL triggering shorter and cut the trajectory into smaller segments. Empirically, shorter T(i) means lower trajectory sinuosity, and makes the approximation more reliable; thus, as shown in Figure 10, when the MP interval is decreased, the CP estimation accuracy of GF scheme is effectively improved.

However, the experiment result shows that when we decrease node density (i.e., increase the MP interval), the performance of the IT scheme seems to be enhanced, which is contrary to our expectations. As far as we know, this phenomenon may be caused by the measuring error of T(i). Actually, when the MP interval is more compact, T(i) is cut into shorter intervals, but the measuring error of T(i) remains unchanged; hence, the T(i) measuring error ratio increases. In this way, the effect of the measuring error of T(i) will gradually become more apparent and cause greater CP estimation error. Nevertheless, according to the performance, the GF scheme obviously has stronger robustness against the T(i) measuring error and performs better under higher LOSL density. Under circumstances of low LOSL densities, when the MP interval is higher than 2.5 m, the IT scheme maintains high CP estimation accuracy under 0.4 m error over all trajectories, which indicates that it has the advantage of robustness to theoretical error, in addition to being able to cope with higher trajectory sinuosity and lower node density in comparison to the GF scheme. 

### 6.3. The CP Estimation Performance versus the MP Position Noise

If the position of MPs is no longer certain, and is assumed to be instantly acquired through active location techniques with estimation error, the CP estimation will surely be deteriorated. In the worst cases, the larger MP estimation deviations will completely distort the ground truth topology, as previously mentioned in Figure 7. Fortunately, the LOSL triggering sequence is measured under the topology of ground truth, which means that it can be used to identify whether the estimated position of MP is qualified for CP estimation (we could even consider using the LOSL triggering sequence in turn to refine the active positioning performance of the MP). 

The experiment is conducted by the hardware-in-the-loop simulation as follows: the triggering sequence and elapsed times T(i) are measured in situ under PATH1 with MP intervals equal to 2, 3, and 4 m; and LM, GF, and IT algorithms are performed assuming that the MP positions are corrupted by additive Gaussian noise with zero-mean and standard deviation σ:

(18) $$\left[x^{\prime }{}_{MP}\;y^{\prime }{}_{MP}\right]=\left[x_{MP}\;y_{MP}\right]+\left[\sigma_{x}\;\sigma_{y}\right],\;\sigma_{x},\sigma_{y}\sim N\left(0,\sigma \right)$$

**Figure 11:**
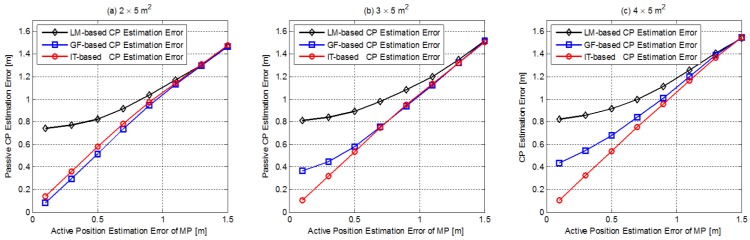
The CP estimation error for the GT schemes versus MP estimation error under different node densities for PATH1.

Abandoning the invalid MP location estimation according to Figure 7, the ERT method is adopted for CP estimation for the IT scheme, and the CP estimation performance via MP positioning deviation is given is Figure 11 after 1000 Monte Carlo ensemble runs.

It is apparent that CP estimation RMSEs are increased with higher MP positioning noise, as expected. The CP estimation error is generally in the same order of magnitude as the MP positioning error. When σ is at low level, the IT scheme performs better than the other two schemes, verifying the feasibility of the ERT algorithm to estimate the CP when the MP position is estimated with deviation. As the MP positioning error rises, performances of the GF and the IT schemes are no longer better than the simplest LM scheme, and all GT schemes gradually perform at the same level. This is because serious MP positioning deviation significantly weakens the efficiency of the GF and IT algorithms; in essence, these two algorithms rely more on the elapsed times accurate topology information, and serious MP positioning deviation undoubtedly distort the measure elapsed times. In addition, the influence of the node density is also weakened when the MP positioning error is at a high level.

## 7. Conclusions and Future Works

In this paper, we focused on utilizing geometric theorems for CP estimation on the LOSLs to track a device-free target under WSN scenarios. As the prerequisite of geometric theorems, we investigated the characteristics of the RF Tomography to distinguish TA from NTA in order to guarantee LOSL detection. Then, we reviewed the existing CP estimation schemes based on geometry theorems, including the LM and the GF algorithms, and proposed a novel CP estimation scheme, adopting the Intercept Theorem. Moreover, considering that MP positioning deviations will distort the AP-MP topology, the ERT method was proposed to estimate the CP by the IT algorithm when the estimated LOSLs are no longer parallel to one another.

Finally, the performance of the GT-based CP estimation was evaluated in four respects: firstly, we compared the requirement of wireless node density of the geometry-based passive tracking schemes with that of other existing RF Tomography-based schemes. The result of this comparison revealed the low hardware requirement advantage of geometric theorem-based schemes. Secondly, we demonstrated the properties of all geometric theorem-based CP estimation schemes, taking into account four aspects including speed requirement, node distribution, computing cost, and real-time performance. Thirdly, the CP estimation accuracy of three geometric theorem-based tracking schemes was evaluated under different LOSL densities, when the device-free target walks in three routes with different levels of tortuosity. The CP estimation under noisy topology was evaluated as the last issue. In general, the evaluation of three geometric theorem-based scheme revealed the following information:

(1) Compared with the existing passive tracking schemes utilizing RF Tomography, the GT-based schemes can achieve decent tracking accuracy under a rather low density of wireless nodes, as shown in Table 1 presented in Section 6.1;
(2) The LM scheme has the lowest accuracy, but it has the advantage of no requirements for prior knowledge of target speed or LOSL distribution. It also has low computing cost and good real-time performance, which enhance its practicability;
(3) The GF scheme and the IT scheme have enhanced tracking performance compared to the LM scheme, although the requirement of speed for the GF scheme and the requirement of parallel LOSL pairs for the IT scheme limit their applicability;
(4) The GF schemes have enhanced performance at higher node density scenarios; meanwhile, it should be noted that the proposed IT scheme can still provide remarkable tracking accuracy even under long interval of MPs over all trajectories, outperforming the other schemes;
(5) When the MP position is mixed with deviation, the IT scheme still works by using the ERT method, and even outperforms the other two schemes under scenarios with low levels of deviation.

The information presented above should be taken into consideration when selecting an appropriate geometric theorem-based passive tracking scheme according to the application circumstance and accuracy requirement.

Our future work will consist of the following three topics: Firstly, to extend the application potential of the GT-based passive tracking schemes in harsher indoor environments, the detection of attenuation action for non-LOS links should be researched, including using a combination of pattern matching algorithms and diversity technologies (spatial diversity and frequency diversity), increasing the operating frequency, and so forth. Secondly, tracking multiple targets is an important and tough issue for device-free target tracking schemes; to the best of our knowledge, the success rate of multiple target tracking work depends on the size of the monitoring area and the target, as well as the node density and distribution topology, which should be appropriately addressed in the near future. Other important future work is the joint active and passive positioning system; as previously mentioned, MPs should be instantly located to track a device-free target. However, the measured information for passive tracking can in turn refine the active positioning performance (as mentioned in Section 6.3). As such, the investigation of how to coordinate active and passive positioning systems will be an interesting and practical topic. Furthermore, the influence of the measurement error of LOSL triggering timestamps on the CP estimation performance should be evaluated as well.

## Figures and Tables

**Table 1:** Comparison of wireless node density and positioning accuracy of various passive tracking schemes.

Schemes	Nodes Number	Monitoring Area	Nodes Density (per m^2^)	Average Error
GT-Based Schemes ^1)^	10	5 ×12 m^2^	0.167 nodes	LM: 0.8 m GF: 0.4 m IT: 0.3 m
Ref. [10]	28	4 × 4 m^2^	1.75 nodes	0.02 m
Ref. [11]	34	9 × 8 m^2^	0.47 nodes	0.45 m
Ref. [15] ^2)^	34	① 5 × 12 m^2^	① 0.61 nodes	① 0.58 m
		② 8 × 9 m^2^	② 0.47 nodes	② 0.90 m
Ref. [17]	24	8 × 8 m^2^	0.375 nodes	0.6 m

Note: ^1)^ Performance of geometric schemes is referred to in Figure 8 (PATH3 and MP interval equals 4 m) in the following section; ^2)^ ① Bookstore Case, ② Office Case.

**Table 2:** Practicability analysis of the GT-based passive tracking schemes.

Schemes	Node Distribution	Pedestrian Speed	Real Time	Computing Cost
LM	Free	Free	Good	Low
GF	Free	Roughly Needed	Fair	Median
IT	Required	Free	Fair	Low

Note: The item “Node Distribution” indicates whether or not the LOSLs need to be distributed parallel to one another.

## Abbreviations

RSS	Received Signal Strength	MP	Monitor Point
WSN	Wireless Sensor Network	CP	Crossing Point
RF	Radio Frequency	LM	Least Mean-square-error
GT	Geometric Theorem	GF	Geometrical Formulation
LOSL	Line-of-Sight Link	IT	Intercept Theorem
AP	Accessing Point	SWM	Sliding Weighted Mean
TA	Traverse Attenuation	ERT	Equal-Ratio Travers
CDF	Cumulative Distribution Function	NTA	Non-Traverse Attenuation
RMSE	Root Mean Square Error	ASL	Approximate Straight Line
